# Using Geospatial Mapping to Determine the Impact of All-Terrain Vehicle Crashes on Both Rural and Urban Communities

**DOI:** 10.5811/westjem.2017.6.34404

**Published:** 2017-07-25

**Authors:** Evelyn S. Qin, Charles A. Jennissen, Caroline A. Wadman, Gerene M. Denning

**Affiliations:** University of Iowa, Carver College of Medicine, Department of Emergency Medicine, Iowa City, Iowa

## Abstract

**Introduction:**

Deaths and injuries from all-terrain vehicle (ATV) crashes result in approximately 700 deaths each year and more than 100,000 emergency department (ED) visits. Common misconceptions about ATV crashes are a significant barrier to injury prevention efforts, as is the lack of key information about where and how crashes occur. The purpose of this study was to determine ATV crash patterns within a state, and to compare and contrast characteristics of these crashes as a function of crash-site rurality.

**Methods:**

We performed descriptive, comparative, and regression analyses using a statewide off-road vehicle crash and injury database (2002–2013). Comparisons were performed by rurality as defined using the Rural Urban Commuting Area (RUCA) coding system, and we used geographic information system (GIS) software to map crash patterns at the zip code and county levels.

**Results:**

ATV crashes occurred throughout the state; 46% occurred in urban and 54% in rural zip code areas. Comparisons of rider and crash characteristics by rurality showed similarities by sex, age, seating position, on vs. off the road, and crash mechanism. Conversely, helmet use was significantly lower among victims of isolated rural crashes as compared to other victims (p=0.004). Crashes in isolated rural and small rural areas accounted for only 39% of all crashes but resulted in 62% of fatalities. In both rural and urban areas, less than one-quarter of roadway injuries were traffic related. Relative crash rates varied by county, and unique patterns were observed for crashes involving youth and roadway riders. During the study period, 10% and 50% of all crashes occurred in 2% and 20% of the state’s counties, respectively.

**Conclusion:**

This study suggests that ATV crashes are a public health concern for both rural and urban communities. However, isolated rural ATV crash victims were less likely to be helmeted, and rural victims were over-represented among fatalities. Traffic was not the major factor in roadway crashes in either rural or urban areas. Unique crash patterns for different riding populations suggest that injury prevention experts and public policy makers should consider the potential impact of geographical location when developing injury prevention interventions.

## INTRODUCTION

Since all-terrain vehicles (ATV) were introduced in the 1970s, U.S. Consumer Product Safety Commission (CPSC) data have shown a significant increase in ATV-related deaths and injuries.^1^ Current estimates indicate that there are approximately 700 deaths each year and more than 100,000 emergency department (ED) visits.^1^,^2^

There are a number of independent risk factors for ATV-related deaths and injuries. These factors include being male, under 16 years of age, inexperience, carrying passengers, alcohol use, and lack of helmets.^3^–^13^ Numerous studies indicate that these unsafe riding practices are highly common.^9^,^14^–^24^ Crash location has also been shown to be associated with the likelihood and severity of injuries. Specifically, deaths are more common on roadways than off, and severe injuries are more likely on the road.^3^,^7^,^25^ Moreover, even after controlling for multiple variables including helmet use, ATV fatality victims in roadway crashes were nearly twice as likely to have suffered a head injury as compared to off-road victims.^5^

Consistent with these outcome results, epidemiologic and survey studies have shown that unsafe riding behaviors are more likely on the roads than off. For example, fatal roadway crashes were more likely than fatal off-road crashes to involve multiple riders and alcohol use, and victims of these fatal roadway crashes were less likely to be helmeted.^5^ Alcohol use and lower helmet use were also found to be more likely in non-fatal roadway crashes as compared to non-fatal crashes off the road.^7^

Similarly, survey studies found a high prevalence of unsafe riding behaviors among adolescent students who had been on an ATV, with 92% reporting having ridden on an ATV with passengers and 81% reporting having ridden on a public road.^16^ Students reporting both riding on the road and carrying passengers had a more than three-fold higher likelihood of reporting having been in a crash. Among adult participants surveyed at a large agricultural event, over 80% had ridden with passengers and two-thirds had ridden on public roads.^14^ Over half of survey respondents reported never or almost never wearing a helmet.

Only two studies have examined the geographic patterns of ATV-related deaths and injuries. A West Virginia study of fatalities found that 20 out of 55 counties (36%) in their state accounted for nearly seven out of 10 fatal crashes from 2000 to 2008.^26^ Another study showed that the eastern region of Texas had a higher ATV-related pediatric (<18 years old) death rate than the state as a whole.^27^ The goals of the current study were to determine the ATV crash patterns in a Midwestern state using an off-road vehicle crash and injury database that combines information from multiple statewide sources, and to determine the extent to which crash site rurality was associated with crash characteristics.

## METHODS

### Off-Road Vehicle Crash and Injury Database

We compiled an off-road vehicle crash and injury database that included records from the Iowa Department of Transportation (DOT), Iowa Department of Natural Resources (DNR) and State Trauma Registry (STR) for the years 2002–2013. The University of Iowa Institutional Review Board approved these studies.

Population Health Research Capsule

What do we already know about this issue?

ATV-related deaths and injuries are an important but often overlooked public health issue. Major vulnerable populations include youth under 16 years old and riders who take their ATV on the road.

What was the research question?

What are the ATV crash patterns within a state and are there differences in crash characteristics between urban and rural areas?

What was the major finding of the study?

Crash patterns differed for vulnerable riding populations and illustrated the need for targeted interventions at the county level.

How does this improve population health?

Injuries are a leading cause of death and disability. Using geospatial mapping to locate ATV crashes provides key information for targeted community-based injury prevention.

### Identifying ATV Crashes

The data sources for the database include more than one off-road vehicle type. To identify ATV crashes for inclusion in this study, we used several strategies. Vehicle type for DOT data was determined using the vehicle identification number (VIN). For DNR data, a vehicle type variable is included on the crash form and was usually documented. In some cases, make and model were also available. For STR data, we used E-Codes for initial identification (821.0–821.9). We then used cause-of-injury narratives to further identify vehicle type. For both DNR and STR data, ATVs were distinguished from side-by-sides (utility task vehicles, UTVs; recreational off-highway vehicles, ROVs) using the make and model when available or by reading all crash narratives for key words describing side-by-side features, i.e., rollover protection structures (ROPS) and seatbelts. Records without sufficient vehicle information were designated as unknown. Only records with vehicle type designated as an ATV were used in the current study. We resolved data for duplicate records in more than one database prior to analysis.

### Study Variables

In analysis, we used variables that were moderately (e.g., crash mechanism, helmet use) to well documented (demographics) in the combined database. Because crash-related variables were coded from the trauma registry narrative, a limited number of these variables had documentation sufficient for inclusion in bivariate and multivariate analysis. Person-related variables used in this study were the victim’s sex, age, seating position, helmet use, and whether the injury was fatal. Crash-related variables used were crash mechanism, whether the crash occurred on or off the road, and rurality of the crash location. Rurality was based on zip codes and was defined using the Rural Urban Commuting Area Codes (RUCA) 2.0 from the University of Washington (http://depts.washington.edu/uwruca/ruca-approx.php). Specifically, we combined the 10 levels in the original coding system into four categories: isolated rural, small rural, large rural, and urban as previously described.^28^

### Mapping Crashes

We used ArcGIS (v10.2) to create visual representations of crash patterns at the zip code and the county level. Point-source mapping and analysis were not feasible as only DOT data provided geographic information system (GIS) coordinates, and DNR and STR data were limited in documentation of street address of the crash site. County and zip code locations were available for 1,832 unique crashes.

We mapped crashes in each county both as total number of crashes over the study period and as crash rate (crashes per 100 registered ATVs). ArcGIS selected cutoff points for the scale to optimize comparisons. The registration data used in the study was made available from the Iowa DNR but did have some limitations. Most importantly, the registrations provided were likely an underestimate of the total number of ATVs in the state both because ATVs used exclusively as farm equipment are not required to be registered and because there is no consistent enforcement of registration for non-occupational use. In addition, prior to 2012 only the number of newly purchased vehicles registered each year was available, not total registrations. So, we used registration data from 2012–2015 in the study. The total number of registered ATVs for 2012–2015 was 30,186, 25,564, 23,856, and 24,020, respectively.

To calculate a crash rate for each county, we divided the number of crashes in the county during the study period by the average number of registered vehicles for the county from 2012–2015. Values were multiplied by a factor of 100 to generate whole numbers. Due to inherent limitations in the ability to capture all ATV crashes in the state and the limitations in registration data, these numbers should be considered best estimates and used as relative rather than as absolute values to compare counties.

To indicate the rurality of the crash location for mapped data, zip code areas were shaded based on RUCA coding, with darker shades indicating more urban areas. Relative crash numbers and crash rates by county are shown as a shaded scale with darker shades representing higher values.

### Data Analysis

We used SPSS (IBM Statistics Package for the Social Sciences, v22) to perform all analyses. Descriptive analysis generated frequencies of study variables, and comparisons of categorical variables were performed using the chi-square test. We used logistic regression analysis to calculate adjusted odds ratios and 95% confidence intervals (CI) for categorical outcomes, after controlling for significant covariates. Persons with missing data for one or more of the variables in the model were not included in analysis. Only helmet use was identified in bivariate analysis as being different by rurality. Thus, helmet use was the only outcome variable used in regression modeling. The number of records with values for all variables in this model was 479.

## RESULTS

### Crash Characteristics

The database contained 2,202 unique ATV crashes involving 2,326 crash victims for the study period. Victims were 78% males and 29% were youth less than 16 years of age (Table 1). Operators were 83% of crash victims and only 25% of all victims were wearing helmets at the time of the crash. Among persons in the database who were injured, 2.6% died.

The major crash mechanism was a non-collision event like a rollover (74%), and less than 10% of all crashes involved a collision with another motorized vehicle. One in four crashes occurred on the road. Even on roadways, however, only 23% of crash victims (101 out of 445) were involved in a traffic collision. Similarly, although more ATV-related fatalities (8 of 56 victims, 14%) than non-fatal injuries (107 of 2176 victims, 5%) resulted from traffic-collisions (p=0.005), still more than eight out of 10 fatalities were from single-vehicle crashes.

**Figure 1:**
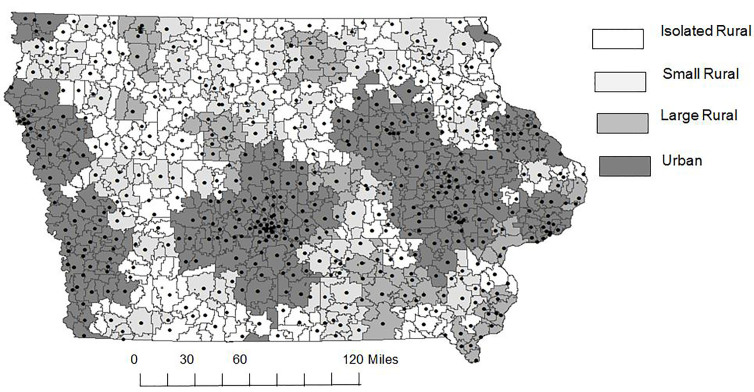
Zip code pattern of all-terrain vehicle (ATV) crashes in Iowa recorded in the Off-Road Vehicle Crash and Injury Database for the years 2002–2013 (n=1,832 crashes). Map shows zip code location of crashes with shading based on the Rural Urban Commuting Area (RUCA) coding system.

Approximately 83% of crashes (1,832 of 2,202) in the database had location information for mapping by the zip code area of the crash site. Figure 1 shows the pattern of crashes in the state with zip code areas shaded by rurality. Mapping showed crashes occurred throughout the state.

### Comparisons by Crash-Site Rurality

Using the RUCA coding system, 46% and 54% of all crashes occurred in urban and rural zip code areas, respectively, with a similar proportion for the three rural designations (Table 1). Comparisons of demographics and crash characteristics by rurality are shown in Table 2.

We observed no significant differences as a function of rurality, except for helmet use. Differences in the proportion of fatal versus non-fatal crashes and for crashes on roadways vs. off-road approached but did not reach significance. Of note, almost three-fourths of fatalities (35 of 49, 71%) were in rural zip codes and over half of all fatal crashes (24 of 43, 56%) occurred on the road.

We used regression analysis to further characterize the potential association of helmet use with rurality and other variables (Table 3). Results indicated that passenger victims, riders in roadway crashes, and crash victims in isolated rural areas were 55%, 61%, and 62% less likely to be helmeted than operators, off-road riders, and crash victims from urban areas, respectively. Consistent with results from bivariate analysis, we saw no differences in likelihood of helmet use by sex or age of the crash victims.

### Crash Patterns by County

**Figure 2:**
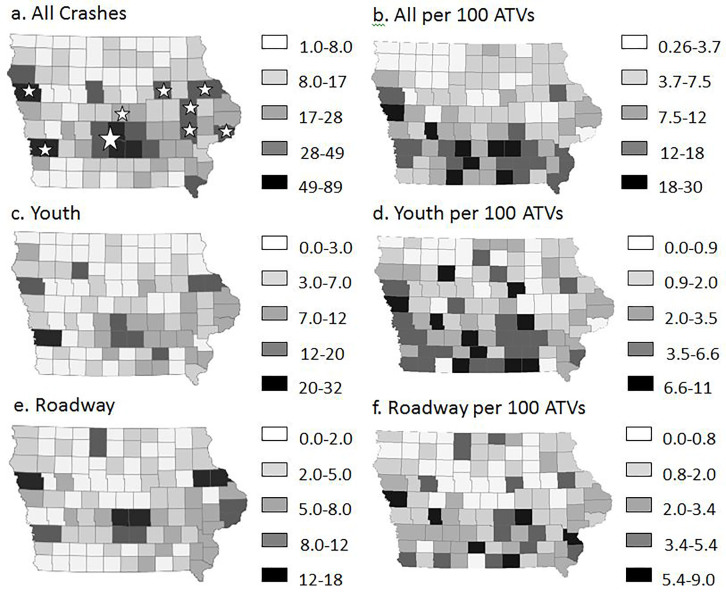
Patterns of all-terrain vehicle (ATV) crashes in the Off-Road Vehicle Crash and Injury Database for the years 2002–2013 by county (n=1,832 crashes). Values in the indicated ranges (automatically selected by ArcGIS for optimal grouping of crashes) are represented using a shaded scale. Crash rates were based on an estimated number of registered vehicles per county and are expressed as crashes per 100 registered ATVs. Panel a, b: Maps show crash number and crash rate for all crashes in each county. Stars represent the largest cities with the larger star representing four cities in the Des Moines metropolitan area. Panel c, d: Maps show crash number and crash rate for crashes in each county involving youth <16 years old. Panel e, f: Maps show crash number and crash rate for roadway crashes in each county.

We mapped total crashes and crash rates per 100 registered ATVs at the county level for all crashes in the database (Figure 2, Panels a, b), for those involving youth less than 16 years old (Figure 2, Panels c, d) and for those that occurred on the road (Figure 2, Panels e, f). Patterns show county-level variability in each case.

With respect to all crashes, the highest numbers were most often observed in counties with major cities. In Figure 2 (Panel a), stars represent the location of the top 12 largest cities in the state. The larger star represents four of these cities that are contiguous. While counties with the highest total crash numbers were primarily in central and eastern parts of the state, areas with the highest crash rates based on registered ATVs were in rural southern counties (Figure 2, Panel b).

As with total crashes, the number of crashes in each county involving youth (Figure 2, Panel c) or on the road (Figure 2, Panel e) was highest near population centers. However, in contrast to data for all crashes, counties with the highest crash rates for youth-related (Figure 2, Panel d) and roadway crashes (Figure 2, Panel f) were more widely distributed throughout the state. The crash patterns by county were also different for these two high-risk riding populations.

Counties were sorted by number of crashes per county for all crashes, for youth-related crashes, and for crashes on the road. We calculated total crashes for the counties with the highest numbers and determined their percentage of total crashes (Table 4). Results showed that in all three cases, 2%, 20%, and 33% of counties accounted for approximately 10%, half, and two-thirds of all crashes, respectively.

## DISCUSSION

### Scope of the Problem

Overall knowledge and public awareness of ATV safety appears to be limited.^14^,^29^,^30^ In addition, survey results of knowledge and safety behaviors show that many riders either do not know what is safe or do not practice safe riding behaviors despite this knowledge.^2^,^9^,^16^,^18^–^20^,^24^,^31^,^32^ The high proportions of ATV crash victims who exhibit unsafe behaviors at the time of the crash is consistent with these survey results.^5^–^7^,^25^ Although previous studies showed that location, on vs. off the road, is associated with differences in riding behaviors and outcomes,^5^,^7^,^25^,^33^ no studies had previously examined associations between rurality of the crash site and ATV-related deaths and injuries. Both similarities and differences between rural and urban areas are informative.

### Rurality and Demographics

Our study showed, for the first time, that helmet use was independently associated with rurality. Specifically, we found that helmet use was significantly lower among crash victims in isolated rural crashes, as compared to victims of crashes in other areas. This finding is consistent with results from a school-based survey study.^16^ Students from school districts in isolated rural areas were less likely to report wearing helmets than their peers in other school districts. In contrast to helmet use, comparisons of other rider characteristics in this study showed no significant differences between riding populations in rural and urban settings.

### Fatal Crashes

Among ATV crash victims in the database, 2.6% were killed. Whereas isolated rural and small rural zip code areas accounted for only 39% of all crashes, 62% of fatal crashes occurred in these areas. The reason for this finding is currently unknown. However, because both the crash mechanism and proportion of roadway crashes were not different by rurality in this study, neither likely account for the higher proportion of fatal crashes in rural areas. Previous studies have shown that rural victims have a higher risk of death from traumatic injury than their urban peers, though the basis for this increased risk also remains elusive.^34^ We speculate that longer response times for emergency medical services to rural crash victims and, in some cases, longer times before more remote crashes are detected may contribute to the differences observed. Lower rates of helmet use among rural crash victims may also be a factor.

### Youth ATV Crashes

Younger age has been identified as an independent risk factor for ATV-related deaths and injuries.^4^ The American Academy of Pediatrics’ policy states that no child under the age of 16 should be allowed on an ATV,^35^, and the Consumer Product Safety Commission and manufacturers warn against youth under 16 years of age riding on adult-size vehicles. Crash rates (per 100 registered ATVs) for youth in the study varied from county to county. These differences suggest more frequent riding by youth in some counties than in others and/or that youth in counties with higher crash rates are more likely to have engaged in risky riding behaviors.

### Roadway Crashes

A commonly held misconception is that ATV riding on public roads is safe. This is not supported by the findings in this and previous studies.^5^,^7^,^25^ Nearly 30% of all crashes and more than half of fatal crashes in the database occurred on the road, and the proportion of roadway crashes was similar for rural and urban areas. Previous studies also showed more than half of all fatal U.S. crashes occurred on the road and that both paved and unpaved roads represented greater risks than riding off-road.^5^,^25^ As with youth-related crashes, the rate of roadway crashes varied by county. This suggests that roadway riding may be occurring to a greater extent in some counties than in others and/or that riders in high crash-rate counties are engaging in risky behaviors (e.g., multiple riders on the ATV) to a greater extent on the road than riders in counties with lower rates.

### Crash Mechanism

We have noted at state and local traffic safety meetings that traffic engineers tend to make the assumption that roadway crashes of other off-road vehicles, e.g., tractors, provide a model for thinking about how to prevent ATV crashes on the road. Directly comparing farm vehicle and ATV roadway crashes, however, demonstrates that this is not the case.

A previous study of roadway farm equipment crashes (not including ATVs) across a nine-state Midwest region found that almost one-third (30%) of crashes occurred in urban RUCA zip codes.^28^ However, a closer look showed that most of these crashes occurred at the interface of rural and urban areas. The current study found that 46% of crashes occurred in urban zip codes, but the pattern showed a relatively broad distribution in both rural and urban areas, with no apparent aggregation at rural-urban interfaces (Figure 1).

There is also a significant difference in crash mechanism between farm vehicles and ATVs. For the former, motor vehicle collisions accounted for nearly 90% of all crashes,^28^ and this may explain in part increased crash rates as vehicles reach rural-urban interfaces. In sharp contrast to these results, approximately three out of every four roadway ATV crashes were not traffic-related. This was true in both rural and urban areas. Thus with respect to crash pattern and mechanism, ATVs and farm vehicles are dramatically different, and rural roads with low traffic density should not be considered “safer” for ATVs than roads/streets in other areas. In fact, as stated earlier, mortality risk is higher in rural areas, possibly due to delayed emergency medical responses.

### Potential Implications for ATV Injury Prevention

If public policy makers or healthcare providers hold the common misconception that ATV crashes are mostly a problem for farm families, then it seems less likely they will perceive ATV injury prevention as a statewide priority. Moreover, because urban areas tend to command more resources than rural ones, this misconception could create a barrier to finding sufficient support for ATV injury-prevention efforts in a state.

Although survey studies for ATVs^16^ and mopeds^36^ previously showed lower reported helmet use among rural vs. urban youth, this is the first study to show that helmet use is independently associated with rurality for ATVs. These data also suggest that lack of a helmet safety culture may be more pronounced in smaller rural communities. Helmet laws remain a critically important issue in public health and would significantly help reduce both fatal and non-fatal traumatic brain injuries from crashes of ATVs and other open motorized vehicles.

There remains a disturbing trend toward counties and cities passing ordinances allowing recreational ATV riding on public roads.^37^ This study provides yet more evidence that roadway riding is dangerous, including on rural roads. Moving forward, it will be important to monitor the extent to which legalizing riding on roadways impacts ATV crashes and injuries using approaches similar to these study methods.

The study identified counties in the state with higher numbers of ATV crashes and with higher relative crash rates. Safety-minded collaborators in these counties could be recruited to develop specific injury prevention programs for those areas. Using this approach may be valuable to other organizations and agencies that wish to determine their statewide crash patterns and to identify vulnerable riding populations and specific regions for which targeted interventions could be developed.

## LIMITATIONS

These studies have the limitations inherent in retrospective research and those experienced by other ATV injury prevention researchers. These limitations include incomplete capture of crash and injury records and/or incomplete variable documentation. The data sources used in this study are more likely to record moderate to serious crashes and injuries, rather than crashes resulting in injuries not requiring medical attention or those that only required medical care in an outpatient clinical setting.

Additionally, because of limitations in trauma registry crash narratives, some side-by-sides (UTVs, ROVs) may have been documented as ATVs and included in the study. Even if true, however, we hypothesize that it did not introduce significant bias in the results, as identified side-by-sides only comprised around 3% of the off-road vehicle crashes in our database.

Whether victims were wearing a helmet was documented in less than half of all cases, largely due to lower documentation in the state trauma registry. We speculate that there may also have been a bias toward documenting whether victims were helmeted (notable fact) vs. not (highly common) in trauma records. If this bias does exist, however, then reported helmet use in this study would be an over-estimate. Of note, helmet use in the study was similar to that seen in other studies, including those using national data.^5^ Moreover, regression analysis demonstrated associations between lack of helmet use and seating position or crash location (on road vs. off) that were also seen previously with national data.^5^ Thus, the finding that helmet use is inversely associated with rurality may be more generalizable.

This study represents a single state. However, it should be noted that demographics and crash characteristics in the study are very similar to those reported by other states and to national data and that all states have rural areas and urban areas similar to those in Iowa.

As outlined in the “Methods” section, caution should be used in interpreting crash rates because of the limitations in ATV registration data and capture of crashes and injuries. However, if one assumes these limitations apply equally across the state, then it seems reasonable to consider values as relative crash rates when comparing counties.

## CONCLUSION

Results from these studies demonstrate that ATV crashes are a public health concern for both rural and urban communities. They further highlight concerns regarding youth on ATVs, low helmet use (particularly in smaller rural communities), and riding on public roads, including those in rural areas. Demographics, location (on vs. off the road), and crash types (collisions vs. non-collisions) did not differ significantly by rurality suggesting that riding populations and riding behaviors are similar across the state. However, variability in crash rates suggests county-based differences in riding frequency and/or unsafe riding behaviors. Approaches used in this study provide a better understanding of where crashes occur, and can help safety advocates identify areas for which injury prevention interventions may be most needed and/or have the greatest impact. These findings may also help the public, as well as city, county and state governments, understand the wider nature of the problem and the need to invest state resources in ATV injury prevention efforts. Similar approaches could be valuable in other states.

## Figures and Tables

**Table 1:** Person and crash related characteristics for ATV crashes in the Iowa Off-Road Vehicle Crash and Injury Database from January 1, 2002 through December 31, 2013.

Variable	n^1^	Col %
Sex		
Male	1809	78%
Female	497	22%
Age		
<6 years old	61	2.6%
6–11 years old	212	9.1%
12–15 years old	384	17%
16–17 years old	192	8.3%
18–30 years old	641	28%
31–45 years old	426	18%
46–60 years old	259	11%
>60 years old	98	4.2%
Seating		
Operator	1386	83%
Passenger	277	17%
Helmet use		
No	935	75%
Yes	311	25%
Fatality		
No	2257	97.4%
Yes	60	2.6%
Roadway crash		
No	1127	75%
Yes	371	25%
Crash mechanism^2^		
ATV-ATV	82	4.4%
ATV-VEH	94	5.0%
ATV-OTHER	307	16%
NON-COLLISION	1385	74%
Rurality		
Isolated rural	313	19%
Small rural	321	20%
Large rural	237	15%
Urban	738	46%

*Col*, column.

1 Column totals (n) for each variable may not equal total n for persons or crashes due to missing data.

2 ATV-ATV, collision between 2 or more ATVs; ATV-VEH, collision of ATV with a motor vehicle that is not another ATV; ATV-OTHER, ATV collision with a fixed or unfixed object that is not a motor vehicle; NON-COLLISION, event did not involve a collision with a motor vehicle or object.

Crashes n=2,202; Victims n=2,326.

**Table 2:** Comparison of victim and crash characteristics as a function of rurality for ATV crashes in the Iowa Off-Road Vehicle Crash and Injury Database from January 1, 2002, through December 31, 2013.

	Rurality (RUCA^1^) n (Column%)^2^				
		
	Isolated rural	Small rural	Large rural	Urban	p value^3^
Person-related variables					
Sex					
Male	296 (79%)	276 (80%)	196 (78%)	608 (77%)	0.68
Female	72 (21%)	68 (20%)	56 (22%)	181 (23%)	
Age					
<6 years old	9 (3%)	13 (4%)	9 (4%)	18 (2%)	0.1
6–11 years old	41 (12%)	25 (7%)	20 (8%)	66 (8%)	
12–15 years old	67 (20%)	59 (17%)	41 (17%)	146 (19%)	
16–17 years old	24 (7%)	24 (7%)	20 (8%)	75 (10%)	
18–30 years old	87 (26%)	97 (29%)	86 (35%)	193 (25%)	
31–45 years old	53 (16%)	58 (17%)	40 (16%)	152 (20%)	
46–60 years old	37 (11%)	42 (12%)	18 (7%)	97 (12%)	
>60 years old	15 (5%)	22 (6%)	13 (5%)	31 (4%)	
Helmet use					
No	156 (83%)	133 (68%)	104 (71%)	338 (68%)	0.004
Yes	28 (17%)	51 (32%)	36 (29%)	136 (32%)	
Seating					
Operator	219 (84%)	204 (82%)	154 (81%)	448 (83%)	0.89
Passenger	42 (16%)	44 (18%)	36 (19%)	95 (17%)	
Fatality					
No	323 (97%)	331 (97%)	245 (99%)	777 (99%)	0.089
Yes	13 (3%)	14 (3%)	8 (1%)	14 (1%)	
Crash-related variables					
Crash mechanism^3^					
ATV-ATV	10 (4%)	11 (4%)	14 (7%)	28 (4%)	0.75
ATV-VEH	15 (5%)	18 (7%)	11 (5%)	48 (8%)	
ATV-OTHER	51 (19%)	42 (16%)	33 (16%)	100 (16%)	
NON-COLLISION	197 (72%)	191 (73%)	146 (72%)	447 (72%)	
Roadway crash					
No	173 (77%)	186 (78%)	149 (78%)	387 (77%)	0.089
Yes	70 (29%)	54 (23%)	42 (22%)	159 (29%)	

1 Rural Urban Commuting Area coding system

2 Column total (n) for each variable may not equal total n due to missing data.

3 Categorical variables were compared using the chi square test.

4 ATV-ATV, collision between 2 or more ATVs; ATV-VEH, collision of ATV with a motor vehicle that is not another ATV; ATV-OTHER, ATV collision with a fixed or unfixed object that is not a motor vehicle; NON-COLLISION, event did not involve a collision with a motor vehicle or object.

Crash n=2,202; Victim n=2,326.

**Table 3:** Likelihood of crash victim being helmeted.^1^ Multivariable regression analysis related to crash-victim helmet use in the Iowa Off-Road Vehicle Crash and Injury Database from January 1, 2002, through December 31, 2013.

Covariates^2^	aOR	95% CI
Sex		
Male	1.15	0.67–1.97
Female	Ref (1.0)	
Age		
< 16 years old	1.38	0.88–2.16
≥ 16 years old	Ref (1.0)	
Seating		
Operator	Ref (1.0)	
Passenger	0.45	0.23–0.88
Roadway		
No	Ref (1.0)	
Yes	0.39	0.24–0.64
Rurality		
Isolated rural	0.38	0.21–0.70
Small rural	0.81	0.49–1.37
Large rural	0.87	0.49–1.55
Urban	Ref (1.0)	

*aOR*, adjusted odds ratio.

1 Reference is not being helmeted.

2 Model included the indicated covariates. Cases missing data for one or more of the variables were not included in the model. Final included cases = 479.

**Table 4:** Proportion of crashes in the state as a function of the counties with the highest number of crashes for each of the indicated crash categories in the Iowa Off-Road Vehicle Crash and Injury Database from January 1, 2002 through December 31, 2013

Crashes (Total n)	Counties^1^ n (%)	Crashes n (%)
All^2^ (n=1,805)	2 (2%)	175 (10%)
	20 (20%)	907 (50%)
	33 (33%)	1196 (66%)
Youth^2^ (n=552)	2 (2%)	52 (9%)
	20 (20%)	272 (49%)
	33 (33%)	369 (67%)
Roadway^2^ (n=424)	2 (2%)	34 (8%)
	20 (20%)	209 (49%)
	33 (33%)	285 (67%)

1 Total number of counties = 99.

2 The counties in order from highest to lowest crash number for the three populations are similar but not identical.
